# Midgut Volvulus: A Rare but Fatal Cause of Abdominal Pain in Pregnancy—How Can We Diagnose and Prevent Mortality?

**DOI:** 10.1155/2020/2185290

**Published:** 2020-05-26

**Authors:** Eelyn Chong, David S Liu, Neil Strugnell, Vishnupriya Rajagopal, Krinal K Mori

**Affiliations:** ^1^Department of Obstetrics and Gynaecology, The Northern Hospital, 185 Copper Street, Epping 3076, VIC, Australia; ^2^Department of General Surgery, The Northern Hospital, 185 Copper Street, Epping 3076, VIC, Australia

## Abstract

Midgut volvulus in pregnancy is rare but life-threatening, resulting in high maternal and fetal mortality. This surgical emergency commonly masquerades as symptoms of pregnancy, which together with its low incidence often leads to delay in diagnosis and definitive treatment. Here, we review the last three decades of the literature, discuss the challenges in managing this rare condition, and raise awareness among clinicians to minimise loss of life.

## 1. Introduction

Bowel obstruction is rare in pregnancy with an approximate incidence of 1 in 10,000 [1]. The main underlying aetiologies include adhesions (60%), volvulus (25%), intussusception (5%), carcinomas (3.5%), and hernia (1.5%) [1].

Volvulus is defined as twisting of a segment of intestine around its own mesentery, which is typically long and narrow-based. This results in extrinsic vascular occlusion and consequent ischaemic infarction of the twisted intestinal segment. In general, intestinal volvulus most commonly affects the sigmoid colon, followed by the caecum, small bowel, and transverse colon [2, 3]. Small bowel or midgut volvulus, which is often ileocolic, accounts for 25% of all intestinal volvulus and is as rare as 1–3% of all cases of bowel obstruction in pregnancy [2, 3]. It is the most feared, as it compromises the superior mesenteric arterial pedicle, leading to extensive loss of small and large bowel and predisposes to short gut syndrome.

Midgut volvulus usually presents with generalised abdominal pain and bilious vomiting [2]. The degree of intestinal volvulus will dictate the tempo of symptomatic onset and the acuity of presentation. As the symptoms of midgut volvulus may appear nonspecific and mimic those of pregnancy, diagnosis and definitive management of midgut volvulus in pregnancy is often delayed, precipitating catastrophic outcome.

Here, we review the last three decades of literature with the aim of discussing the management approaches for pregnant women presenting with midgut volvulus.

## 2. Methodology

**Figure 1:**
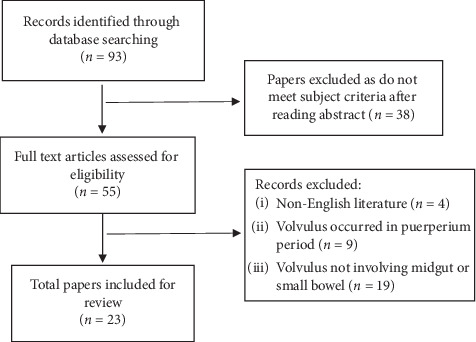
Search strategy.

A comprehensive literature search using keywords “midgut volvulus”, “small bowel volvulus”, and “pregnancy” was performed via MEDLINE® and PubMed databases with time period between year 1990 and 2019. Selected articles were then obtained in full text and reviewed for suitability by two independent reviewers (EC and DL). Only patients with midgut or small bowel volvulus in pregnancy were considered for review, excluding those who were diagnosed during the puerperium. A full diagram of the search strategy is provided in Figure 1.

## 3. Discussion

In the past 29 years, only 23 cases of midgut volvulus have been published. As shown in Tables 1 and 2, common predisposing factors for volvulus include adhesions from previous surgeries and underlying congenital malrotation. Midgut volvulus typically presents in the second and third trimesters. This phenomenon may be explained by several factors. First, a rapidly enlarging gravid uterus displaces the anatomical location of intra-abdominal viscera [10]. Second, relaxin release during pregnancy increases tissue pliability [2]. Both factors may thus predispose to midgut volvulus in already susceptible individuals such as those with congenital malrotation or adhesions [2].

The maternal and fetal outcomes following maternal midgut volvulus can be disastrous, especially if the diagnosis is delayed. Overall, our review demonstrated that maternal and fetal mortality was 13% and 35%, respectively. We also observed that all maternal deaths occurred in the third trimester. We postulate that volvulus, in an anatomically predisposed patient, intermittently occurs and resolves in the nonpregnant patient or early gravid patient. However, during the third trimester of pregnancy when there is an increased uterine height and size, predisposed patients may experience a static barrier, which prevents resolution of the volvulus, leading to a mechanical closed loop obstruction with development of venous infarction. It is known that fetal outcomes are directly linked to maternal physiology [2, 10]; hence, delayed diagnosis of midgut volvulus may lead to bowel infarction with hypovolaemia, renal failure, and septic shock that result in fetal compromise.

The classic triad of midgut volvulus consists of generalised abdominal pain, vomiting, and obstipation, which overlap with common symptoms during pregnancy [2]. During pregnancy, uterine enlargement gradually displaces the bowel into the epigastrium rendering the signs of volvulus atypical. In late pregnancy, the abdominal pain of volvulus usually transitions from colicky to constant in nature. It is mostly felt in the epigastrium. This should be differentiated from the paroxysmal pain of uterine contraction [2]. New onset back pain may also suggest intra-abdominal pathology [8]. Meticulous history taking to elicit the nature of vomiting is important as the presence of bilious content indicates small bowel obstruction, which should prompt further investigation. Due to the hyperdynamic circulatory state of pregnancy, patients with midgut volvulus do not necessarily present in the first instance with shock. Fever, tachycardia, and leucocytosis are often late signs in pregnancy and manifest when the involved bowel has infarcted [2, 3]. Therefore, in an obstetric patient with an unremarkable medical history, presenting with abdominal pain, bilious vomiting, and obstipation, one should consider surgical causes in addition to obstetric or gynaecological aetiologies. Importantly, normal biochemistry does not exclude midgut volvulus [22]. Serial and frequent observations with bedside and blood tests are essential.

Early diagnosis relies on sound clinical assessment and effective use of radiology. A hesitation to pursue radiological investigations in pregnancy is often the main barrier in achieving a definite diagnosis. The maximum radiation dose that a fetus can be safely exposed to is 10 rads. Currently no single diagnostic study exceeds 5 rads [6, 18]. Pregnant women with a suspected acute abdomen should be informed about the safety of radiological imaging. Ultrasonography (US) and magnetic resonance imaging (MRI) have been reported to be safe in pregnancy with no associated risk to the fetus [18]. US is often used first line; however, the displacement of intra-abdominal viscera with the gravid uterus can limit its sensitivity [18]. MRI plays an important role in diagnosing volvulus with the characteristic ‘whirlpool sign' demonstrating mesenteric torsion in addition to closed loop obstruction with transition points [2, 18]. Modalities that rely on ionizing radiation such as abdominal X-ray (AXR) and computed tomography (CT) have also been reported. Evidence of dilated small bowel with multiple air-fluid levels on AXR should heighten the suspicion of intestinal obstruction, although these are not always diagnostic [4, 5, 7]. Low-dose CT of the abdomen and pelvis is also an option when other tests are inconclusive as this is thought to be the most appropriate imaging modality to evaluate for mesenteric ischaemia in the general population [1, 23]. It is important to take into account the accessibility and availability of the imaging tool as this should not delay surgery if bowel obstruction is clinically suspected in a pregnant woman with a virgin abdomen. Interestingly, despite being the most readily available form of radiological imaging, AXR is not frequently used when pregnant women presented with symptoms of midgut volvulus according to our literature review. This is likely due to the fear of radiation exposure as mentioned earlier.

If bowel obstruction is suspected in pregnant women, a proactive approach to management should be undertaken with aggressive IV fluid hydration, nasogastric decompression, and electrolyte replacement [2, 15]. Not infrequently, the underlying aetiology may not be apparent after clinical assessments and further investigations. Rapid and multidisciplinary surgical intervention improves the patient's chance of survival. Among the 23 cases, the average duration from symptom onset to diagnosis was 56 hours. In one case report, a patient with massive midgut volvulus was only diagnosed 26 hours after the development of her symptoms and underwent extensive small and large bowel resection but unfortunately passed away later due to complications from short gut syndrome [5].

The definitive management of midgut volvulus is almost always surgery. In our review, only two cases did not involve adhesiolysis and/or bowel resection [14, 15]. One was managed conservatively with anticoagulation in the setting of superior mesenteric thrombosis, and the other was managed endoscopically with a nasojejunal tube in the second trimester. Both cases had good maternal and fetal outcomes. The severity of bowel ischaemia determines the extent of surgical intervention for midgut volvulus. In cases where all intestines are still viable, detorsion of the volvulus and restoration of normal anatomy, such as Ladd's procedure may be sufficient [22]. In the presence of infarction, resection is mandatory. Whether anastomosis is performed primarily or as a two-stage procedure depends on the patient's physiology. Short gut syndrome is a recognised complication of extensive bowel resection, in which the patient would require life-long total parenteral nutrition. This alone is associated with significant short- and long-term morbidity [5]. In one case report, the patient was palliated after an exploratory laparotomy that revealed extensive intestinal infarction [13].

An important issue is the impact of bowel resection on future fertility. Data on the ideal time interval between pregnancies after extensive bowel resection are scarce; therefore, we cannot make an evidence-based recommendation. However, we suggest that nutritional optimisation following bowel resection has been achieved before considering further pregnancy.

## 4. Conclusion

There is currently a limited understanding and thus a lack of consensus regarding the optimal management of midgut volvulus during pregnancy. Nonetheless, awareness of this rare but life-threatening condition will form the basis for meticulous clinical assessment, which when supported by judicious use of radiological investigations will hopefully minimise delay in diagnosis and treatment. Timely surgical intervention in a multidisciplinary manner is necessary to prevent loss of life.

## Figures and Tables

**Table 1:** Cases of midgut volvulus in pregnancy (excluding puerperium) from 1990–2019.

Authors, year	Age (year)	Gestation (weeks)	Symptom duration	Method of diagnosis	Aetiology	Treatment	Maternal outcome (alive/deceased)	Foetal outcome (alive/demised)
Wax and Christie[4]	31	24	7 days	AXR	Adhesions from previous surgery	Adhesiolysis, no bowel resection	Alive	Demised
Matthews and Soper [3]	18	23	8 days	Surgery	Congenital gut malrotation	Small and large bowel resection	Alive	Demised
Kusnetzoff et al. [5]	30	35	1 day	AXR	Superior mesenteric thrombosis	Bowel resection and stoma	Deceased	Demised
Wheeler et al. [6]	29	28	ND	Surgery	ND	Bowel resection and anastomosis	Alive	Demised
Damore et al. [7]	27	26	>7 days	AXR	Congenital gut malrotation	Adhesiolysis, appendectomy	Alive	Alive
Ventura-Braswell et al. [8]	22	37	>2 days	Surgery	Congenital gut malrotation	Bowel resection and anastomosis	Alive	Alive
Dilbaz et al. [9]	19	32	1 day	US + surgery	ND	Bowel resection and anastomosis	Alive	Alive
Biswas et al. [10]	20	31	>4 days	CT	Adhesions from previous surgery	Bowel resection and anastomosis	Alive	Alive
Mahdavi and Yunesi [11]	20	10	>2 days	Surgery	ND	Bowel resection and anastomosis	Alive	Demised
Kuwahata et al. [12]	32	39	4 days	CT	Adhesions from previous surgery	Bowel resection and anastomosis	Alive	Alive
Gaikwad et al. [13]	27	33	ND	CT	Superior mesenteric occlusion	Exploratory laparotomy, palliation	Deceased	Demised
Shui et al. [14]	25	35	4 days	Surgery	Superior mesenteric thrombosis	Anticoagulation, no bowel resection	Alive	Alive
Siwatch et al. [15]	23	20	>2 days	CT	Congenital gut malrotation	Endoscopic decompression	Alive	Alive
Vassiliou et al. [16]	35	21	2 days	MRI	ND	Bowel resection and anastomosis	Alive	Alive
Sharma et al. [17]	28	9	3 days	Surgery	Congenital gut malrotation	Adhesiolysis, no bowel resection	Alive	Alive
Kouki et al. [18]	34	14	ND	MRI	Congenital gut malrotation	ND	ND	ND
Nameirakpam et al. [19]	35	32	2 days	Surgery	ND	Bowel resection and anastomosis	Alive	Demised
Hwang et al. [20]	22	38	9 hours	Surgery	Congenital gut malrotation	Bowel resection	Deceased	Alive
Cong et al. [2]	26	37	8 hours	Surgery	Adhesions from previous surgery	Adhesiolysis, no bowel resection	Alive	Alive
Webster et al. [1]	30	39	1 day	CT	Adhesions from previous surgery	Adhesiolysis, no bowel resection	Alive	Demised
Constanthin and Darouichi [21]	29	28	2 days	MRI	Adhesions from previous surgery	Adhesiolysis, no bowel resection	Alive	Alive
Antunes et al. [22]	38	27	ND	MRI	Congenital gut malrotation	Ladd's procedure	Alive	Alive
Esterson et al. [23]	28	33	2 days	CT	Congenital gut malrotation	Adhesiolysis, no bowel resection	Alive	Alive

AXR: abdominal X-ray; CT: computed tomography; MRI: magnetic resonance imaging; US: ultrasound; ND: not described.

**Table 2:** Summary of midgut volvulus by trimester (1990–2019).

Trimester	Cases (n)	Most used method of diagnosis	Maternal mortality	Fetal mortality
1 (1–12 weeks)	2	Surgery (*n* = 2)	0%	50% (*n* = 1)
2 (13–28 weeks)	10	MRI (*n* = 3)	0%	30% (*n* = 3)
3 (29–40 weeks)	11	Surgery/CT (*n* = 5 each)	25% (*n* = 3)	36% (*n* = 4)
